# Clinical characteristics and risk factors for a prolonged length of stay of patients with asymptomatic and mild COVID-19 during the wave of Omicron from Shanghai, China

**DOI:** 10.1186/s12879-022-07935-w

**Published:** 2022-12-16

**Authors:** Chen Hu, Yu-Kai Liu, Qi-Di Sun, Zheng Du, Yu-Qiang Fang, Fei Guo, Yu-Bo Wang, Yong He, Yuan Cen, Fan Zeng

**Affiliations:** 1 Department of Respiratory Disease, Daping Hospital, Army Medical University, Chongqing, China; 2 Department of Cardiology, Daping Hospital, Army Medical University, Chongqing, China; 3 Department of Medical Education, Daping Hospital, Army Medical University, Chongqing, China; 4 Department of Intensive Care Unit, Daping Hospital, Army Medical University, Chongqing, China; 5 Department of Orthopedics, Daping Hospital, Army Medical University, Changjiang Branch Road 10, Chongqing, 400042 China; 6 Department of Neurology and Center for Clinical Neuroscience, Daping Hospital, Army Medical University, Changjiang Branch Road 10, Chongqing, 400042 China; 7 National Exhibition and Convention Center Makeshift Hospital, Shanghai, China

**Keywords:** COVID-19, Omicron, Clinical characteristic, Length of stay, Risk factor

## Abstract

**Background:**

This study aims to investigate the clinical characteristics and the length of hospital stay (LOS), as well as risk factors for prolonged LOS in a cohort of asymptomatic and mild COVID-19 patients infected with the Omicron variant.

**Methods:**

A total of 1166 COVID-19 patients discharged from the inpatient ward of the largest makeshift hospital (May 8–10, 2022) in Shanghai, China, were included. The demographics, medical history, and the lowest and admission cycle threshold (Ct) values of the RT-PCR tests for SARS-CoV-2 genes of the open reading frame 1ab (Ct-ORF) and the nucleocapsid protein (Ct-N) during hospitalization were recorded. Patients with LOS > 7 days, or LOS ≤ 7 days were included in the Prolonged group or the Control group, separately. The clinical characteristics and LOS of the participants in the two groups were described and compared. Multivariate Logistic and linear regression analyses were applied to explore the risk factors for prolonged LOS. The diagnostic efficacy of the lowest and admission Ct values for the Prolonged group was tested via the receiver operating characteristic (ROC) curve analysis.

**Results:**

The median LOS was 6 days in the total study population. The age was older (45.52 ± 14.78 vs. 42.54 ± 15.30, *P* = 0.001), while both the lowest and admission Ct-ORF (27.68 ± 3.88 vs. 37.00 ± 4.62, *P* < 0.001; 30.48 ± 5.03 vs. 37.79 ± 3.81, *P* < 0.001) and Ct-N (25.79 ± 3.60 vs. 36.06 ± 5.39, *P* < 0.001; 28.71 ± 4.95 vs. 36.95 ± 4.59, *P* < 0.001) values were significantly lower in the Prolonged group. There were more mild cases in the Prolonged group (23.8% vs. 11.5%, *P* < 0.001). The symptom spectrum differed between the two groups. In multivariate analyses, age, disease category, and the lowest Ct-N values were shown to be associated with prolonged LOS. Besides, both the lowest and admission Ct-ORF (AUC = 0.911 and 0.873) and Ct-N (AUC = 0.912 and 0.874) showed robust diagnostic efficacy for prolonged LOS.

**Conclusions:**

Our study firstly reports the clinical characteristics and risk factors for prolonged LOS during the wave of the Omicron epidemic in Shanghai, China. These findings provide evidence for the early identification of asymptomatic and mild COVID-19 patients at a high risk of prolonged hospitalization who may require early intervention, and long-term monitoring and management.

**Supplementary Information:**

The online version contains supplementary material available at 10.1186/s12879-022-07935-w.

## Background

By 11 November 2022, there have been 630,832,131 confirmed cases of coronavirus disease 2019 (COVID-19), including 6,584,104 deaths worldwide (https://covid19.who.int/). The pandemic of this infectious disease has impacted global health persistently since December 2019. As first identified in South Africa in November 2021, Omicron, a variant of severe acute respiratory syndrome coronavirus 2 (SARS-CoV-2), was designated as a “variant of concern (VOC)” by the World Health Organization. It has an unusually large number of mutations in the spike proteins mainly located at the receptor-binding domain, which contributes to immune escape and higher infectivity [1, 2]. Moreover, it was reported to escape the neutralization of antibodies, including most therapeutic monoclonal antibodies and vaccine-elicited antibodies [3]. Thus, Omicron has reignited the disease burden with surging global concerns and alertness.

Shanghai, one of the municipalities in China, has been suffering from the wave of the Omicron epidemic since March 2022. Based on the “dynamic-zero” strategy of fighting against COVID-19 in China, a large amount of district-level and municipal-level makeshift hospitals were constructed for the quarantine and management of asymptomatic and mild COVID-19 patients. However, the characteristics of this group of patients remain largely unknown. A few studies on COVID-19 patients during the Omicron wave showed that their median length of hospital stay (LOS) was 3–3.2 days [4, 5]. Meanwhile, age, more severe clinical symptoms, non-vaccination, comorbidities, and low cycle threshold (Ct) values of the RT-PCR tests were shown to be associated with prolonged viral clearance and LOS in patients infected with previous variants [6–10]. Since Omicron is the dominant disease-causing variant at present and the majority of Omicron-infected COVID-19 patients were reported to be asymptomatic and mild cases [11–15], this study aims to demonstrate the clinical characteristics and LOS in a cohort of asymptomatic and mild COVID-19 patients during the wave of the Omicron epidemic in Shanghai from the largest municipal-level makeshift hospital with a capacity of 50,000 beds, the National Exhibition and Convention Center (NECC) Makeshift Hospital, and to investigate the risk factors for the prolonged LOS due to prolonged duration of viral shedding.

## Materials and methods

### Study design and participants

The study population was included from the patients diagnosed with mild or asymptomatic COVID-19 according to Diagnosis and Treatment Protocol for COVID-19 patients (Trial Version 9) [13], which was released by the National Health Commission of China & National Administration of Traditional Chinese Medicine from the NECC makeshift hospital in Shanghai, China. Among them, mild cases were defined as patients with mild symptoms and without abnormalities on chest CT, and asymptomatic cases were defined as patients with positive reverse transcriptase-polymerase chain reaction (RT-PCR) results and without any newly developed clinical symptoms, according to the same protocol. Each admitted patient received one RT-PCR test with the oropharyngeal swab specimen every morning during hospitalization. The inpatients would be discharged if they (1) had two consecutive negative RT-PCR findings at least 24 h apart, without any restrictions on the total LOS; (2) had relieved clinical symptoms if any. The patients discharged from May 8 to May 10, 2022, were consecutively included. Since the average LOS of the 151,018 patients discharged before May 8, 2022 (admission started on April 7, 2022) from the same hospital was 7.03 days (released by the information center of the hospital), the patients with a LOS of more than 7 days (> 7 days) were included in the Prolonged group, and those with a LOS of no more than 7 days (≤ 7 days) were included in the Control group. Patients were excluded if they (1) had severe and complex comorbidities that may cause unspecific symptoms and affect viral shedding, including malignant tumors, immunosuppression, mental disorders, hematological diseases, and chronic hepatic and renal dysfunction [16–21]; (2) failed to be discharged due to any personal reasons when the discharge criteria were met (e.g., staying for taking care of their relatives in the same hospital, etc.). This study was approved by the Institutional Review Board of the Daping Hospital of Army Medical University (2022-165). The obtainment of informed consent from each participant was waived by the board due to the nature of the study. All methods were performed following the Ethical Review of Biomedical Research Involving Human Beings issued by the National Health and Family Planning Commission of China.

### Data collection

The demographics (age, sex, ethnicity), dates of admission and discharge, disease category (mild or asymptomatic), clinical symptoms related to COVID-19, the medical history including hypertension, diabetes, and other self-reported comorbidities, as well as the history of allergy, vaccination (none or incomplete, regular vaccination, and booster vaccination), were recorded. The lowest and admission Ct values of the RT-PCR tests for SARS-CoV-2 genes of the open reading frame 1ab (Ct-ORF) and the nucleocapsid protein (Ct-N) of each participant during hospitalization, considered representative values of indicating the viral load [22, 23], were also recorded. All data were directly obtained from the electronic medical records.

### Statistical analysis

The normal distribution of continuous variables was tested by normality tests, and each continuous variable was shown as mean ± SD (standard deviation) or median (interquartile ranges, IQR) as appropriate. Categorical variables were summarized as the counts and percentages for each category. The differences in age, sex, proportions of mild cases, comorbidities of hypertension and diabetes, the history of allergy and vaccination, the lowest and admission Ct-ORF and Ct-N, as well as the differences in the symptom spectrum between the Prolonged group and Control group, were compared using t-test, Mann–Whitney U test, and Chi-square/Fisher’s exact test as appropriate. To further explore the risk factors associated with the prolonged LOS, multivariate Logistic and linear regression analyses were applied with the forward stepwise (conditional) and enter methods, respectively. The multicollinearity of all covariates was examined using the collinearity analysis. The diagnostic accuracy of the lowest and admission Ct-ORF and Ct-N for LOS > 7 was determined via the receiver operating characteristic (ROC) curve analysis in a nonparametric approach. All analyses were conducted with PASW version 18.0 for windows (SPSS, Inc., Chicago, IL). All hypothesis testing was two-sided, and *P* < 0.05 was considered statistically significant.

## Results

### Demographics and characteristics of the participants

A total of 1188 COVID-19 patients discharged from the NECC makeshift hospital from May 8 to May 10 were firstly screened. Of these, four and nine patients were excluded because of incomplete data and a history of severe and complex comorbidities, respectively. Another nine patients were further excluded because they had met the discharge criteria during hospitalization yet failed to be discharged due to personal reasons. Then 1166 patients were finally included, consisting of 416 (35.7%) patients in the Prolonged group and 750 (64.3%) patients in the Control group. The median LOS of the total study sample was 6 days (IQR = 5 days). There were no differences in age, proportions of male and mild cases between the 151,018 patients discharged 1 month before and the finally included 1166 patients (see Additional file 1).

The median LOS of the Prolonged and Control group was 10 days (4.00 days) and 4 days (2.00 days), respectively. The age was higher (45.52 ± 14.79 vs. 42.54 ± 15.30, *P* = 0.001), while both the lowest and admission Ct-ORF (27.68 ± 3.88 vs. 37.00 ± 4.62, *P* < 0.001; 30.48 ± 5.03 vs. 37.79 ± 3.81, *P* < 0.001) and Ct-N (25.79 ± 3.60 vs. 36.06 ± 5.39, *P* < 0.001; 28.71 ± 4.95 vs. 36.95 ± 4.59, *P* < 0.001) values were significantly lower in the Prolonged group. Besides, there were more mild cases in the Prolonged group (23.8% vs. 11.5%, *P* < 0.001). There were no differences in the history of hypertension (9.1% vs. 7.6%, *P* = 0.359) and diabetes (2.4% vs. 3.7%, *P* = 0.221), the history of vaccination (*P* = 0.089) and allergy (0.5% vs. 1.6%, *P* = 0.157), and the proportions of male (50.0% vs. 47.1%, *P* = 0.337) and ethnic Han patients (98.8% vs. 98.0%, *P* = 0.315) between the two groups (Table 1).

**Table 1:** Demographic and clinical characteristics of the study population

Variables	Prolonged group (n = 416)	Control group (n = 750)	*P* values
Length of stay (days), median (IQR)	10 (4.00)	4 (2.00)	< 0.001
Age (years), mean ± SD	45.52 ± 14.78	42.54 ± 15.30	0.001
Male, (n%)	208 (50.0%)	353 (47.1%)	0.337
Ethnic Han patients, (n%)	411 (98.8%)	735 (98.0%)	0.315
Disease category			< 0.001
Mild patients, (n%)	99 (23.8%)	86 (11.5%)	
Asymptomatic patients, (n%)	317 (76.2%)	664 (88.5%)	
Comorbidity, (n%)			
Hypertension	38 (9.1%)	57 (7.6%)	0.359
Diabetes	10 (2.4%)	28 (3.7%)	0.221
History of allergy, (n%)	2 (0.5%)	12 (1.6%)	0.157^a^
Vaccination, (n%)			0.089
None/incomplete	109 (26.2%)	216 (28.8%)	
Routine vaccination	125 (30.0%)	255 (34.0%)	
Booster vaccination	182 (43.8%)	279 (37.2%)	
Admission Ct values, mean ± SD			
Ct-ORF	30.48 ± 5.03	37.79 ± 3.81	< 0.001
Ct-N	28.71 ± 4.95	36.95 ± 4.59	< 0.001
Lowest Ct values, mean ± SD			
Ct-ORF	27.68 ± 3.88	37.00 ± 4.62	< 0.001
Ct-N	25.79 ± 3.60	36.06 ± 5.39	< 0.001

*SD* standard deviation, *IQR* interquartile range, *Ct* cycle threshold

^a^Fisher’s exact test was applied

### Differences in the symptom spectrum between the two groups

As shown in Table 2, the symptom spectrum between the two groups differed, suggested by higher proportions of patients with fever (29.3% vs. 14.0%, *P* = 0.012), fatigue (33.3% vs. 17.4%, *P* = 0.014), myalgia (24.2% vs. 12.8%, *P* = 0.047), while a lower proportion of patients with stuffiness (9.1% vs. 19.8%, *P* = 0.037) in the Prolonged group. The results were consistent with the finding that there were more mild cases in the Prolonged group, indicating disease severity may be associated with LOS.

**Table 2:** Differences in the symptom spectrum between mild COVID-19 patients in the two groups

Symptoms	Mild cases in the Prolonged group (n = 99)	Mild cases in the Control group (n = 86)	*P* values
Fever, (n%)	29 (29.3%)	12 (14.0%)	0.012
Fatigue, (n%)	33 (33.3%)	15 (17.4%)	0.014
Cough, (n%)	68 (68.7%)	55 (64.0%)	0.496
Sputum, (n%)	32 (32.3%)	31 (36.0%)	0.594
Stuffiness, (n%)	9 (9.1%)	17 (19.8%)	0.037
Rhinorrhea, (n%)	9 (9.1%)	15 (17.4%)	0.092
Throat pain, (n%)	30 (30.3%)	28 (32.6%)	0.742
Myalgia, (n%)	24 (24.2%)	11 (12.8%)	0.047
Hyposmia/hypogeusia, (n%)	3 (3.0%)	6 (7.0%)	0.307^a^
Eye discomfort, (n%)	0 (0%)	1 (1.2%)	0.465^a^
Diarrhea, (n%)	0 (0%)	0 (0%)	NA

*NA* not available

^a^Fisher’s exact test was applied

### Risk factors for the LOS

The risk factors for the LOS were further analyzed via multivariate Logistic and linear regression. The collinearity analysis showed no multicollinearity of covariates including age, sex, ethnicity, disease category, comorbidities of hypertension and diabetes, and the history of allergy and vaccination, except for the four Ct values (see Additional file 2). Therefore, only the two Ct-N values (relatively lower than Ct-ORF in the mean value) along with all the rest covariates were included in two separate regression analyses. The results of multivariate Logistic regression analyses showed that (1) the lowest Ct-N values were associated with a decreased risk of prolonged LOS (OR = 0.707, 95% CI 0.680–0.735, *P* < 0.001); (2) the admission Ct-N values (OR = 0.753, 95% CI 0.730–0.777, *P* < 0.001) and disease category (OR = 1.758, 95% CI 1.164–2.656, *P* < 0.001) were associated with the risk of prolonged LOS. In multivariate linear regression, the lowest Ct-N values (β = − 0.714, *P* < 0.001), age (β = 0.078, *P* = 0.011), and disease category (β = 0.056, *P* = 0.006) were associated with LOS in the total study population (Table 3), consistent with the association of the admission Ct-N values (β = − 0.557, *P* < 0.001), age (β = 0.074, *P* = 0.004), and disease category (β = 0.096, *P* < 0.001) with LOS (Table 4).

**Table 3:** Multivariate linear regression analysis of risk factors for the length of stay of the total study population (The lowest Ct-N values)

Risk factors	B	SE	β	t values	*P* values	95% CI	Collinearity statistics	
							Tolerance	VIF
Lowest Ct-N values	− 0.374	0.011	− 0.714	− 34.806	< 0.001	− 0.395 ~ − 0.353	0.933	1.072
Age	0.013	0.005	0.055	2.556	0.011	0.003 ~ 0.023	0.859	1.165
Sex (male)	− 0.257	0.145	− 0.035	− 1.774	0.076	− 0.541 ~ 0.027	0.985	1.015
Ethnic (ethnic Han)	− 0.153	0.558	− 0.005	− 0.274	0.784	− 1.248 ~ 0.942	0.981	1.019
Disease category (asymptomatic)	0.556	0.203	0.056	2.736	0.006	0.157 ~ 0.954	0.935	1.069
History of allergy	− 0.130	0.665	− 0.004	− 0.196	0.845	− 1.434 ~ 1.174	0.983	1.017
Vaccination (none/incomplete)	0.044	0.089	0.010	0.491	0.624	− 0.131 ~ 0.219	0.979	1.021
Hypertension	− 0.095	0.289	− 0.007	− 0.328	0.743	− 0.663 ~ 0.473	0.822	1.217
Diabetes	− 0.598	0.437	− 0.029	− 1.368	0.171	− 1.456 ~ 0.260	0.855	1.170
(Constant)	18.434	0.465	NA	39.680	< 0.001	17.302 ~ 20.157	NA	NA

The comparator is listed in parentheses after each categorical variable

*B* unstandardized coefficient, *SE* standard error, *β* standardized coefficient, *CI* confidence interval, *VIF* variance inflation factor, *NA* not available

**Table 4:** Multivariate linear regression analysis of risk factors for the length of stay of the total study population (The admission Ct-N values)

Risk factors	B	SE	β	t values	*P* values	95% CI	Collinearity statistics	
							Tolerance	VIF
Admission Ct-N values	− 0.328	0.014	− 0.557	− 22.878	< 0.001	− 0.326 ~ − 0.300	0.935	1.070
Age	0.018	0.006	0.074	2.913	0.004	0.006 ~ 0.030	0.856	1.169
Sex (male)	− 0.336	0.172	− 0.046	− 1.958	0.051	− 0.673 ~ 0.001	0.986	1.014
Ethnic (ethnic Han)	− 0.713	0.662	− 0.026	− 1.077	0.282	− 2.012 ~ 0.586	0.983	1.018
Disease category (asymptomatic)	0.952	0.240	0.096	3.960	< 0.001	0.480 ~ 1.424	0.941	1.062
History of allergy	0.229	0.789	0.007	0.289	0.772	− 1.320 ~ 1.777	0.982	1.018
Vaccination (none/incomplete)	0.009	0.106	0.002	0.080	0.936	− 0.200 ~ 0.217	0.978	1.023
Hypertension	− 0.078	0.344	− 0.006	− 0.228	0.820	− 0.752 ~ 0.596	0.822	1.217
Diabetes	− 0.964	0.519	− 0.047	− 1.858	0.063	− 1.982 ~ 0.054	0.856	1.168
(Constant)	17.285	0.619	NA	27.907	< 0.001	16.070 ~ 18.501	NA	NA

The comparator is listed in parentheses after each categorical variable

*B* unstandardized coefficient, *SE* standard error, *β* standardized coefficient, *CI* confidence interval, *VIF* variance inflation factor, *NA* not available

### The diagnostic efficacy of the lowest Ct values for the prolonged LOS

**Fig. 1:**
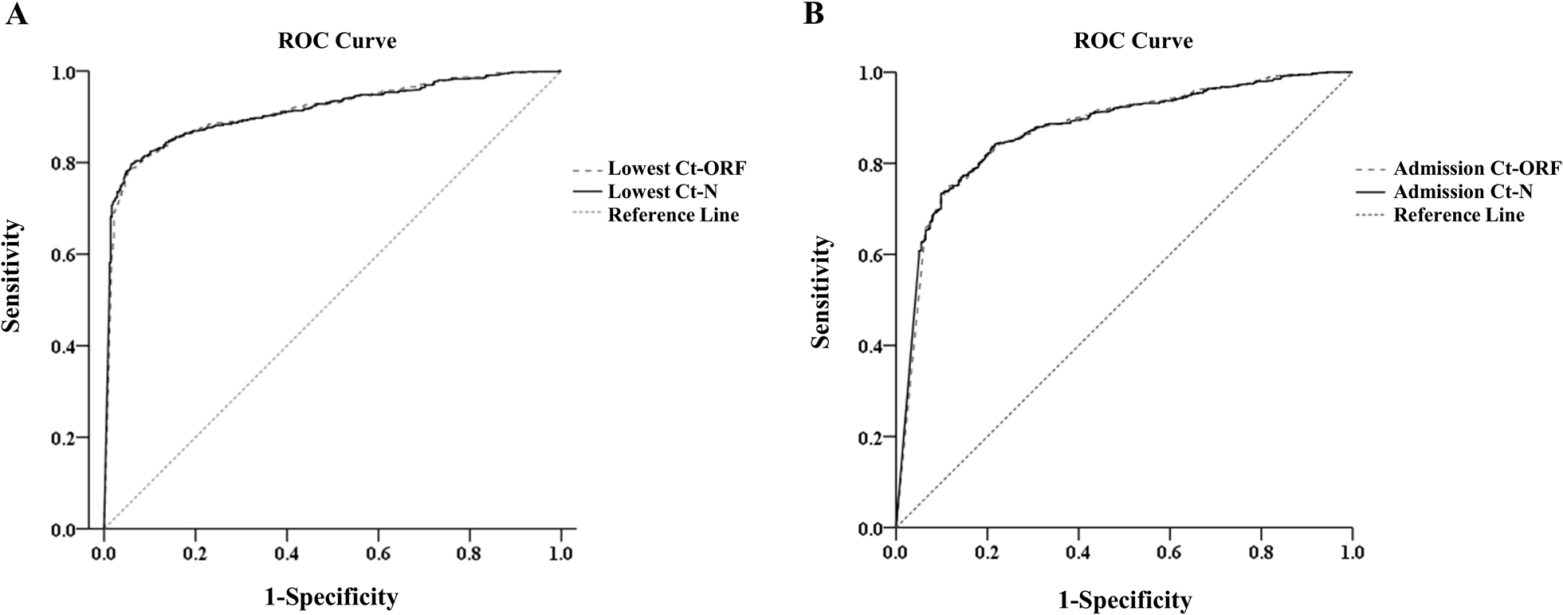
The diagnostic efficacy of the lowest and admission Ct values for prolonged LOS. **A** The results of the ROC analysis showed that the AUC of the lowest Ct-ORF and Ct-N values were 0.911 (*P* < 0.001, 95% CI 0.895–0.928) and 0.912 (*P* < 0.001, 95% CI 0.896–0.929), respectively. **B** The results of the ROC analysis showed that the AUC of the admission Ct-ORF and Ct-N values were 0.873 (*P* < 0.001, 95% CI 0.852–0.894) and 0.874 (*P* < 0.001, 95% CI 0.853–0.895), respectively. *Ct* cycle threshold, *LOS* length of stay, *ROC* receiver operating characteristic, *AUC* area under curve, *CI* confidence interval

Receiver operating characteristic (ROC) analysis was conducted to determine the diagnostic efficacy of the lowest Ct values in distinguishing the Prolonged group from the Control group. The results of ROC analyses showed that the area under curve (AUC) of the lowest Ct-ORF and Ct-N values in the Prolonged versus Control group was 0.911 (*P* < 0.001, 95% CI 0.895–0.928) and 0.912 (*P* < 0.001, 95% CI 0.896–0.929) (Fig. 1A), both slightly higher than those of the admission Ct-ORF (AUC = 0.873, *P* < 0.001, 95% CI 0.852–0.894) and Ct-N values (AUC = 0.874, *P* < 0.001, 95% CI 0.853–0.895) (Fig. 1B). Further Youden index analysis showed that the optimal cutoff value for the lowest Ct-N value was 31.065, corresponded to a sensitivity of 79.7% and a specificity of 94.0%. Similarly, the optimal cutoff value for the lowest Ct-ORF value was 33.420, corresponded to a sensitivity of 78.5% and a specificity of 94.7%.

## Discussion

To the best of our knowledge, this is the first study on the LOS and its risk factors in COVID-19 patients in China during the wave of the Omicron epidemic. According to our findings, asymptomatic COVID-19 patients account for 84.1% of the patients in the makeshift hospital in China. The symptom spectrum differed between patients in the Prolonged group and the Control group. The median LOS of the patients was 6 days (IQR = 5 days). Age, disease category, and both the lowest and admission Ct values of RT-PCR tests during hospitalization were associated with prolonged LOS.

The LOS of COVID-19 patients should be extensively studied owing to its value for optimizing medical resources. A systematic review demonstrated that the median LOS of COVID-19 patients was 14 (IQR 9) days in China, compared with 5 (IQR 6) days outside of China [24]. It was estimated that the LOS for the overall hospital stay of patients not admitted to ICU was 8.0–9.1 days according to the national- and hospital-level data in the UK [25]. However, all these data came from studies on the patients infected with ancestral variants of SARS-CoV-2. A recent study on the characteristics and outcomes of hospitalized patients in South Africa during the Omicron wave showed that the LOS was 3 (IQR 3) days, shorter than the previous waves of ancestral, Beta, and Delta variants [5]. Similarly, another large-sample study on COVID-19 patients caused by the Omicron variant in the US showed that the median LOS of discharged patients was 3.2 days [4]. In our study population, the median LOS was 6 days. The relative longer LOS of COVID-19 patients in China may be explained by differences in criteria for admission and discharge between countries, and different timing within the pandemic. Of note, due to the distinct policies pursued in different countries/areas, asymptomatic and mild patients may not need hospitalization, or two consecutive negative RT-PCR findings (at least 24 h apart) to be discharged outside of China, to which attention should be paid when interpreting the results. However, it is reported that there were less severe COVID-19 cases caused by Omicron variant compared with other SARS-CoV-2 variants, and the majority of Omicron-infected patients were asymptomatic and mild cases [11–15]. Since Omicron is the dominant disease-causing variant of SARS-CoV-2 at present globally, studies on asymptomatic and mild COVID-19 patients are valuable and meaningful. Moreover, the prolonged LOS represents prolonged viral clearance and persistent infectiousness in our study. It may also facilitate the decision-making for the monitoring and management of COVID-19 patients infected with the Omicron variant to investigate the risk factors for prolonged LOS, even in other countries with different admission and quarantine policies. Meanwhile, the LOS of Omicron-infected patients was generally shorter compared with that in studies on previous waves, following the same trend in and outside China. This may be attributed to the previous exposure to SARS-CoV-2, vaccination of the population, and less pathogenicity of Omicron than early variants [5, 26]. This evidence may help the individualization of de-isolation decisions and optimize the utilization of medical resources during the COVID-19 pandemic.

The results of our study showed that the lower Ct values during hospitalization, either the lowest or the admission ones, were the risk factors most significantly associated with the prolonged LOS in both multivariate Logistic and linear regression. And both the lowest and admission Ct-ORF and Ct-N showed robust diagnostic efficacy for prolonged LOS. The Ct values of RT-PCR tests for SARS-CoV-2 are the most acknowledged and convenient indicator for viral load in COVID-19 patients at present [27, 28]. The low Ct values were shown to be associated with delayed clearance of viral RNA in patients infected with previous SARS-CoV-2 variants [8, 9], and our findings further supported that it could be the same case in Omicron-infected patients. A recent study showed that the symptomatic COVID-19 cases across each variant, including Omicron, had higher viral loads than the asymptomatic cases [22]. Besides, the larger number of clinical symptoms and lower Ct values were shown to be associated with a longer LOS and slower viral clearance [6, 8], which were consistent with the longer LOS in mild cases compared with asymptomatic ones in this study. Age was another important factor previously reported to be associated with a worse outcome, including disease progression, in-hospital mortality, as well as prolonged duration of viral shedding, in COVID-19 patients [28–32]. Older age was also associated with longer LOS in Omicron-infected patients in our study. Aside from the overall impaired immune system and heavier comorbidity burden, it may partially be attributed to a lower routine/booster vaccination rate in the older patients (61.1% in participants aged ≥ 60 vs. 74.3% in participants aged < 60, *P* < 0.001, data not shown), considering the evidence that considering the evidence that vaccination can contribute to virus clearance and disease attenuation in patients infected with previous variants [7] as well as the Omicron variant [33]. Since the therapy of nirmatrelvir plus ritonavir (or Paxlovid), recommended by WHO for non-severe COVID-19 patients at a high admission risk [34], was associated with reduced risks of hospitalization, mortality and in-hospital disease progression [35, 36], it is worthwhile to further validate whether patients with low Ct values would benefit from early antiviral treatment.

The study has several limitations. First, patients’ virus genotyping was unavailable. However, the origin of this wave of COVID-19 epidemic was reported to come from the imported cases, and Omicron BA.2 and BA.2.2 variants were detected as the predominant strains announced by Shanghai Municipal Health Commission. Second, only asymptomatic and mild patients were included in this study. Although the disease severity due to the Omicron infection cannot be fully determined based on the limited data at present, it is believed that this variant is milder in pathogenicity compared with the other VOCs [26, 37–39]. Therefore, the study population consisting of asymptomatic and mild COVID-19 patients may represent the majority of the Omicron-infected cases. Third, patients with severe and complex comorbidities were excluded from this study, considering their potential effects on causing unspecific symptoms and viral shedding. Fourth, due to the limited medical equipment in makeshift hospitals, no laboratory or radiological examinations were done in our cohort. Future studies with more information on these data and a larger sample size are necessary to confirm our findings. Finally, although the present study failed to reveal the association between vaccination and LOS, whether different vaccines can shorten the LOS of Omicron-infected COVID-19 patients still need to be validated in the future.

## Conclusion

In conclusion, our study firstly reports that the median LOS of COVID-19 patients in the makeshift hospital was 6 days during the wave of the Omicron epidemic in Shanghai, China. Age, disease category, and the lowest and admission Ct values of RT-PCR tests were significantly associated with prolonged LOS. These findings provide evidence for the early identification of asymptomatic and mild COVID-19 patients at a high risk of prolonged hospitalization who may require early intervention, and long-term monitoring and management.

## Supplementary Information

**Additional file 1.** The comparison of the basic demographic and clinical characteristics between the study population and patients discharged 1 month before.

**Additional file 2.** The collinearity analysis of all covariates in the multivariate Logistic regression.

## Data Availability

The datasets used and/or analysed during the current study available from the corresponding author on reasonable request.
