# Supplementary material for: Clinical characteristics and risk factors for a prolonged length of stay of patients with asymptomatic and mild COVID-19 during the wave of Omicron from Shanghai, China
Source: BMC Infect Dis. 2022 Dec 16;22:947. doi: 10.1186/s12879-022-07935-w (PMC9756685; doi:10.1186/s12879-022-07935-w)
Supplement: Supplementary file 1 — Additional file 1. The comparison of the basic demographic and clinical characteristics between the study population and patients discharged 1 month before. [file 12879_2022_7935_MOESM1_ESM.docx]

**Additional file**[**1**](https://bmcinfectdis.biomedcentral.com/articles/10.1186/s12879-022-07820-6#MOESM1)**. The comparison of the basic demographic and clinical characteristics between the study population and patients discharged one month before**

| Variables | Study population  (n=1166) | Patients discharged 1m before  (n=151,018) | *P* values |
| --- | --- | --- | --- |
| Age (years), mean ± SD | 43.61±15.18 | 42.85±15.29 | 0.09^a^ |
| Male, (n%) | 561 (48.11%) | 78630 (52.10%) | 0.07^b^ |
| Disease category |  |  | 0.33^b^ |
| Mild patients, (n%) | 185 (15.87%) | 22408 (14.84%) |  |
| Asymptomatic patients, (n%) | 981 (84.13%) | 128610 (85.16%) |  |

^a^ Student's t-test was applied.

^b^ Chi-square test was applied.

Abbreviations: SD, standard deviation; 1m, one month.
