# Supplementary material for: Clinical characteristics and risk factors for a prolonged length of stay of patients with asymptomatic and mild COVID-19 during the wave of Omicron from Shanghai, China
Source: BMC Infect Dis. 2022 Dec 16;22:947. doi: 10.1186/s12879-022-07935-w (PMC9756685; doi:10.1186/s12879-022-07935-w)
Supplement: Supplementary file 2 — Additional file 2. The collinearity analysis of all covariates in the multivariate Logistic regression. [file 12879_2022_7935_MOESM2_ESM.docx]

**Additional file**[**2**](https://bmcinfectdis.biomedcentral.com/articles/10.1186/s12879-022-07820-6#MOESM1)**. The collinearity analysis of all covariates in the multivariate Logistic regression**

| Covariates | Collinearity Statistics | |
| --- | --- | --- |
|  | Tolerance | Variance Inflation Factor |
| Age | 0.855 | 1.170 |
| Sex | 0.977 | 1.024 |
| Ethnic | 0.979 | 1.022 |
| Disease category | 0.934 | 1.071 |
| History of allergy | 0.980 | 1.020 |
| Vaccination | 0.975 | 1.026 |
| Hypertension | 0.819 | 1.221 |
| Diabetes | 0.841 | 1.189 |
| Admission Ct-ORF values | 0.042 | 23.934 |
| Admission Ct-N values | 0.034 | 29.457 |
| Lowest Ct-ORF values | 0.018 | 55.466 |
| Lowest Ct-N values | 0.016 | 61.352 |
